# Emotional anticipation after delivery – a longitudinal neuroimaging study of the postpartum period

**DOI:** 10.1038/s41598-017-00146-3

**Published:** 2017-03-08

**Authors:** Malin Gingnell, Simone Toffoletto, Johan Wikström, Jonas Engman, Elin Bannbers, Erika Comasco, Inger Sundström-Poromaa

**Affiliations:** 1 Department of Women’s and Children’s Health, Uppsala University, Uppsala, Sweden; 2 Department of Psychology, Uppsala University, Uppsala, Sweden; 3 Department of Neuroscience, Uppsala University, Uppsala, Sweden; 4 Department of Radiology, Uppsala University, Uppsala, Sweden

## Abstract

Neuroimaging research has begun to unveil the mechanisms behind emotion processing during the postpartum period, which, in turn, may be of relevance for the development of postpartum depression. The present study sought to longitudinally investigate the neural correlates of emotion anticipation during the postpartum period in healthy women. Functional magnetic resonance imaging was employed to measure the blood oxygen level-dependent signal in the brain in response to anticipation of negative emotional stimuli and during processing of images with positive or negative valence. The participating women were scanned twice: the first scan occurred during the first 48 hours after delivery, and the second was performed 4–6 weeks after delivery. The early postpartum period was characterized by higher anterior cingulate cortex reactivity during anticipation of negative emotional stimuli than the late postpartum period. This was accompanied by a negative relationship with insular reactivity during the early postpartum period and a trend towards an increase in insular reactivity in the late postpartum period. Thus, during the first four weeks of the postpartum period, a diminished top-down regulatory feedback on emotion-related areas of the brain was noted. This finding suggests a physiologically important adaptation during the healthy postpartum period.

## Introduction

During the first four weeks of the postpartum period, women experience a multitude of physical and environmental changes and are at risk of developing new-onset postpartum depression or experiencing a worsening of underlying affective disorders^1–4^. These first few weeks following delivery are characterized by a rapid decrease in circulating hormone levels, increased stress, disrupted sleep patterns and altered immune system functioning^4^. Furthermore, because of the increased risk of psychiatric admissions^3^, this period is maintained as a relevant onset specifier in the new DSM-5 criteria of peripartum depression^5^.

During this period, the way the brain handles emotion processing seems to be altered, and, potentially, changes in emotion processing may precipitate affective symptomatology. For instance, healthy postpartum women display enhanced brain reactivity to emotional stimuli^6^, whereas women with postpartum depression (PPD) display attenuated activation within the corticolimbic salience/fear network during emotion processing^6^. Partly in line with this type of reasoning, we recently provided preliminary evidence that healthy women during the first four weeks postpartum develop increased emotion-induced insular and inferior frontal gyrus (IFG) reactivity, with a correlation between brain reactivity and measures of depression and anxiety^7^. Thus, these findings are suggestive of an adaptive process across the first few postpartum weeks that is associated with emotional symptoms experienced by postpartum women. In the present study, we sought to deepen the knowledge of emotion processing and its adaptive changes during the healthy postpartum period by neuroimaging women during anticipated emotion processing longitudinally throughout the first postpartum weeks.

Anticipation of undesirable events is a critical component of emotion processing and is especially relevant for anxiety^8^. A moderate level of anxious anticipation is thought to be adaptive as it allows individuals to prepare for negative events, but excessive anticipatory fear may lead to incapacitating disruption of daily life^8^. Thus, knowledge of the neural substrates of emotion anticipation in healthy postpartum women may facilitate our understanding of the biological mechanisms that predispose women in the postpartum period to clinical depression or anxiety. Among the sparse data at hand, startle response modulation during emotional anticipation has been reported to be reduced from pregnancy to the postpartum period in healthy women^9^, indicating that the anticipatory process is indeed affected in the postpartum period.

Emotion processing is known to involve the amygdala, insula, and the anterior cingulate cortex (ACC), as well as the dorsolateral prefrontal cortex (dlPFC), the medial prefrontal cortex (mPFC), and the orbitofrontal cortex (OFC)^10–12^. In addition to these areas, emotion anticipation also activates more prefrontal areas^13^. Here, an anticipatory task, also involving emotional processing, which has previously been shown to activate prefrontal regions such as the ACC and Brodmann areas (BA) 6, 8, 9 and 10^13^, was employed to study fronto-cingulate reactivity immediately following delivery (early postpartum) and again within four to six weeks postpartum (late postpartum).

Based on the previous reports of reduced affective regulation during emotion anticipation^9^ and increased brain reactivity during emotion processing in the postpartum period^7^, we hypothesized that decreased reactivity in regulatory prefrontal areas and an increased reactivity in the areas more related to emotion processing would be noted at the late postpartum assessment.

## Results

### Participants

The mean age of participating women was 32.6 ± 4.6 years, and the majority had a university education (n = 9, 81.2%). All women were married or cohabitating, and five (45.5%) were primiparous. The participants were scanned twice: the first time was 30.6 ± 8.7 hours after their delivery, and the second scan was performed 33.5 ± 5.4 days after delivery. At the time of the second scan, all women were breastfeeding. Six (54.5%) of the women had a vaginal delivery, and the remaining seven women had a Caesarean section. None of the women were smokers.

As expected, serum concentrations of estradiol and progesterone decreased from the first to the second time point (estradiol: early postpartum 1542 ± 787 pmol/l vs. late postpartum 120 ± 59.9 pmol/l, p < 0.05; progesterone: early postpartum 44.4 ± 37.4 nmol/l vs. late postpartum 0.7 ± 0.5 nmol/l, p < 0.05).

### Anticipation of negative emotional stimuli and emotion processing during the postpartum period

**Figure 1:**
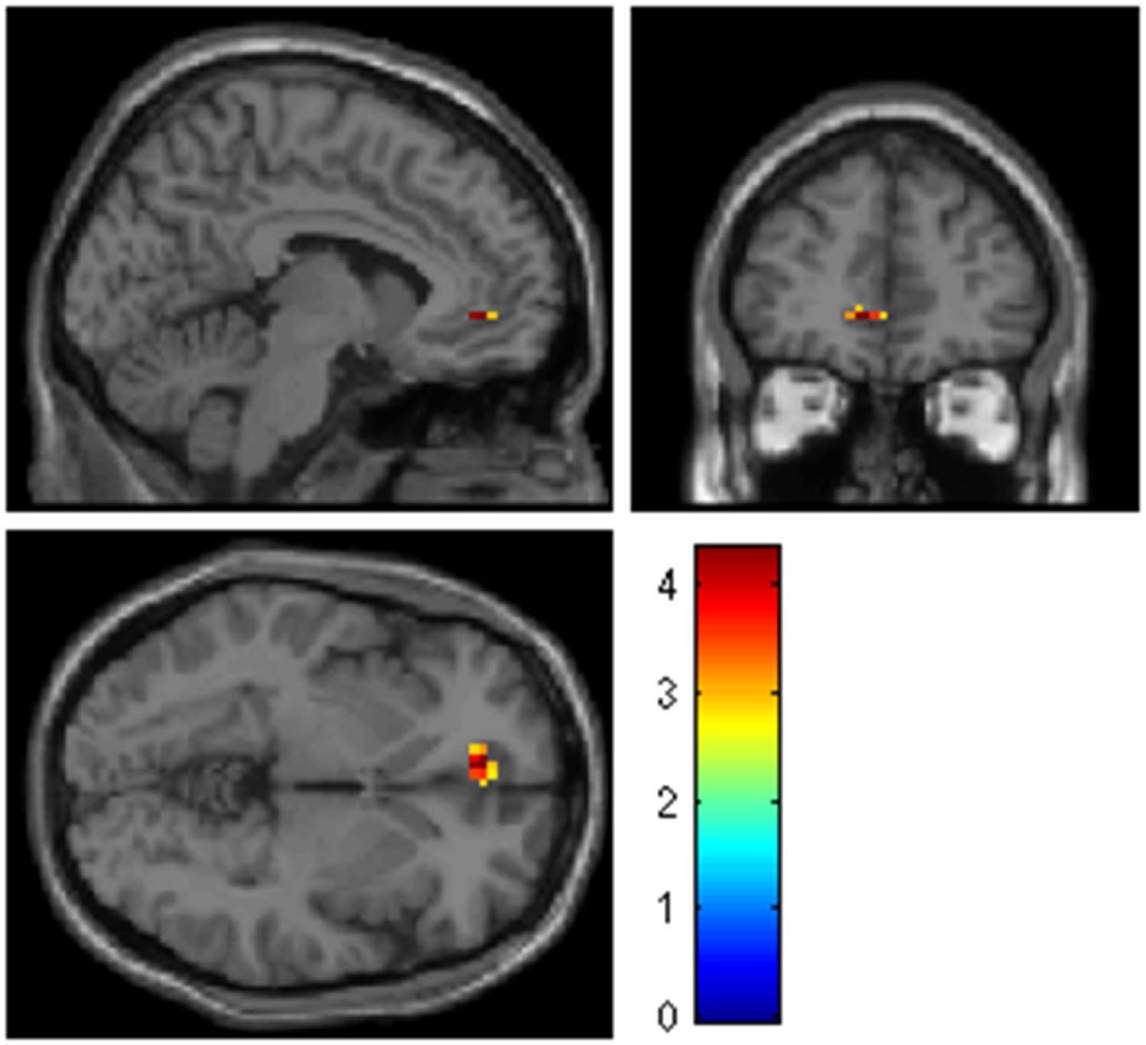
During anticipation of negative emotional stimuli, a higher reactivity was observed in the ACC at the early postpartum assessment than at the late postpartum assessment. The color bar indicates the *T*-scores.

**Figure 2:**
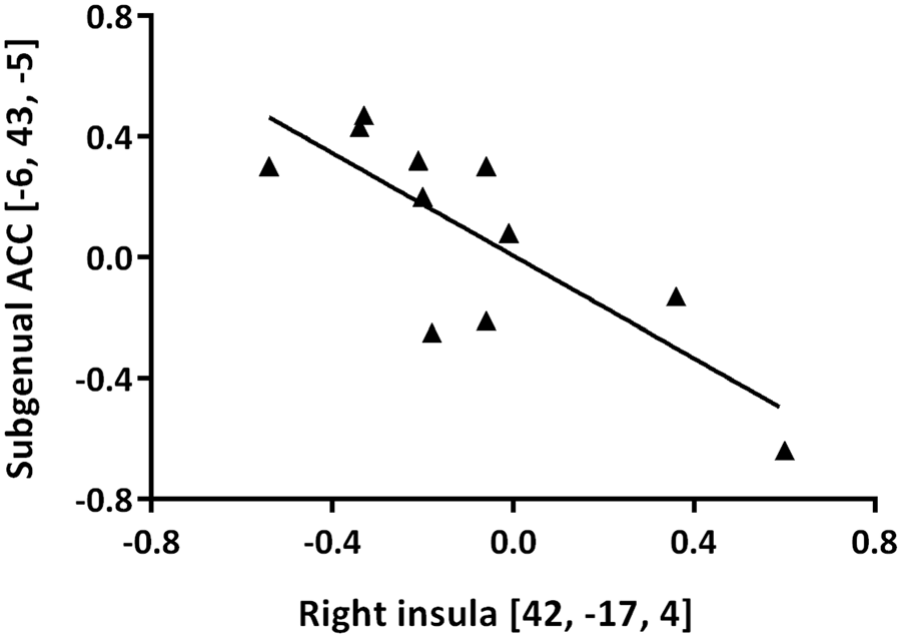
Reactivity in the subgenual ACC was negatively correlated with reactivity in the posterior insula at the early postpartum assessment.

The neural correlates of negative emotional anticipation differed between the early and late postpartum assessments. Increased reactivity was noted in a cluster corresponding to the ACC (BA 32) when women were scanned immediately after delivery in comparison with their later postpartum assessment (Fig. 1, Table 1). Furthermore, at the early postpartum time point, seed-based connectivity suggested a negative coupling between this cluster and the right insula (42, −17, 4; k = 26, z = 3.11, *p *= 0.001, Fig. 2).

**Table 1:** Differences between the early (within 48 h) and late postpartum (after 4–6 weeks) period in blood oxygen level–dependent reactivity during anticipation of negative emotional stimuli and viewing of emotional stimuli, N = 11.

Contrasts	BA	Hemisphere	Cluster size	*Z* score	Talairach coordinates			*p* ^*^
					*x*	*y*	*z*	
**Anticipation of negative images vs. positive images**								
*early postpartum* > *late postpartum*								
	32	L	13	3.17	−6	43	−5	0.001
*late postpartum* > *early postpartum*								
	No significant cluster							
**Viewing of negative vs. positive images**								
*early postpartum* > *late postpartum*								
	32	R	37	3.27	3	41	6	0.001
	24	R		2.89	6	33	12	0.002
	10	R		3.06	3	53	6	0.006
	10	L	38	2.97	−3	55	−2	0.002
	32	L		2.89	−6	47	−2	0.002
*late postpartum* > *early postpartum*								
	No significant cluster							

^*^Corrected for multiple comparisons across the search volume of the region of interest with an extent threshold cluster size ≥10.

Similarly, the neural correlates of emotion processing, i.e., during image viewing, differed between the early and late postpartum assessment. Again, increased reactivity was noted when women were scanned immediately after delivery, where a cluster extending over the ACC (BA 32 and 24) and BA 10 displayed higher reactivity at the early postpartum assessment than the late postpartum assessment (Table 1). However, during emotion processing, there was also a trend for a higher reactivity in the insula at the late postpartum assessment (x/y/z = 39/27/12; z = 3.23, *p *= 0.001, k = 8, BA13).

There were no correlations between the change in BOLD-reactivity across the two postpartum assessments and the corresponding decrease in estradiol or progesterone (data not shown).

## Discussion

We sought to investigate emotion regulation and processing in healthy women across the first few weeks of the postpartum period. By employing fMRI, we studied emotion anticipation and processing in healthy women within two days of delivery and again four to six weeks after delivery. Based on our previous results indicating a reduced regulatory^9^ and increased affective^7^ responsivity by the end of the first postpartum month, we hypothesized that we would observe an adaptive response at the second assessment, consisting of increased reactivity to emotion stimuli, simultaneously with blunted top-down emotional regulatory activity during emotion anticipation. Indeed, anticipation of negative emotional stimuli was associated with greater fronto-cingulate reactivity during the early relative to the late postpartum assessment, and the parts of the ACC where the reactivity changes were observed were within the suggested areas for emotional regulation^10, 14^. Thus, this finding suggests diminished top-down regulatory feedback as the postpartum period progresses. In addition, the observed negative connectivity between ACC reactivity and insular reactivity during emotion anticipation immediately following delivery lend further support to this hypothesis. Presumably, this finding represents a physiologically important adaptation during the healthy postpartum period.

The endogenous and exogenous environment affect emotion processing, and emotion processing, together with cognitive functioning, modulates the responses of an individual^15^. Cognition and emotion are both dependent on one another. During the postpartum period, the constant cognitive challenge, manifested by caretaking of the child and the attachment procedure, might lead to maladaptive responses to emotional stimuli. Thus, the observed blunted emotional regulatory activity during the late postpartum assessment might reflect this. Because the present study did not contain any measures of attachment or maternal behavior, the exact association with maternal behavior and attachment remain speculative. However, it is possible that the reduced ACC reactivity noted four to six weeks postpartum is linked to changes in the emotionality of relevance for maternal behavior or attachment, which at the same time increases the risk for PPD^16^. Due to the selection bias introduced by the rather challenging study design in which fMRI scans are required in close connection to childbirth, the present sample included extremely healthy women with regards to emotional symptoms. For this reason, further investigations in clinical samples are needed.

Several studies have indicated that variations in ovarian steroid levels affect brain reactivity during emotion processing, but we found no evidence for an ovarian steroid-influence on emotion regulation or processing in the postpartum period^17, 18^. Clearly, the postpartum period involves changes not only in ovarian steroid levels but also in γ-Aminobutyric acid^19^-active progesterone metabolites, the hypothalamus-pituitary-adrenal axis, and the oxytocinergic system^4, 20^. In addition, the postpartum period is characterized by both attenuated serotonergic activity^21–24^ and decreased cortical GABA concentrations^25^. Of interest to the present study, women with postpartum depression have been shown to have downregulated serotonergic receptors in the subgenual ACC compared to that in healthy controls^26^. It is possible that in susceptible women, the presumably normal downregulation of emotional inhibition via the subgenual ACC, as observed in the present study by the end of the first postpartum month, increases the risk of depression when it is added to (trait- or state-related) deficits in the serotonergic tonus of the subgenual ACC.

Our earlier observation of increased insula reactivity to emotional stimuli in the late postpartum period^7^ was only partly confirmed within a small cluster in the right insula. This may be due to the use of different paradigms; we previously used a contrast between recognition of emotional faces and simple geometrical shapes and instead applied a more fine-tuned difference between negative and positive visual stimuli in the present study.

Among the methodological strengths of the present study are the longitudinal design and the investigation of a mechanism-oriented fMRI task to dissect emotion regulation. However, among the limitations, the sample size must be mentioned, which calls for independent replication. However, to recruit women within 48 hours of delivery, as well as to engage them in repeated assessments over time, is challenging. It should also be noted that generalization of our findings to newly delivered women may be hampered by some characteristics of our study participants: these women were highly motivated, well-educated, and had physically and psychologically uncomplicated deliveries and postpartum periods. Finally, while the present study used non-specific emotional stimuli, it may still be that different emotion anticipation patterns would be observed in the presence of infant-related visual stimuli^6^.

In conclusion, this study indicates that brain reactivity during anticipation of negative stimuli is altered across the first few weeks of the postpartum period. Using partly the same sample, we have previously shown that insular reactivity during emotion recognition was higher in the late postpartum period^7^ and that prefrontal reactivity during response inhibition decreased throughout the postpartum period^27^. Taken together, these studies indicate a relative attenuation of regulatory activity and an upregulation of emotional reactivity by the end of the first postpartum month, which may affect the risk for PPD.

## Materials and Methods

### Participants

The participating women have been described in detail elsewhere^7^. Briefly, twenty-six right-handed, healthy postpartum women with uncomplicated pregnancies and deliveries were included from the Maternity ward of the Department of Obstetrics and Gynecology, Uppsala University Hospital. Women were approached for participation in the study as soon as possible following delivery but had to have had at least one night of sleep following the delivery. Women were between 18 and 45 years of age, had no ongoing depressive or anxiety disorders according to the MINI International Neuropsychiatric Interview^28^, and had not used any psychotropic drugs within three months prior to delivery. In addition, they had no contraindications to magnetic resonance imaging (MRI), neurological disorders or previous brain trauma.

All women were scheduled to participate in two functional MRI (fMRI) sessions: the first session was within 48 hours after delivery (early postpartum), and the second one was at 4–6 weeks after delivery (late postpartum). At both sessions, women were interviewed about obstetric and neonatal complications, current medication, and breast feeding. The baby was taken care of by the father or a research assistant. Blood samples for hormonal analyses were drawn 20 minutes prior to each scanning session.

Of the twenty-six postpartum women included, eight could not be examined within the 48-hour time-frame due to lack of access to the fMRI equipment. One participant dropped out of the study after the first test session, and three additional participants were excluded: two women because of claustrophobic symptoms while in the scanner, and one for medical reasons (nausea). Two participants were excluded due to incomplete scanning sessions due to hardware problems. Finally, one participant was excluded from the fMRI analyses due to movement artifacts (peaks of movement in the x/y/z-axis of more than 3 mm or more than 2 degrees of rotation). Thus, the final sample consisted of 11 women with complete measures at both the early and late postpartum assessment.

All participants provided written informed consent prior to inclusion, and the procedures were approved by the Regional Ethical Review Board, Uppsala, Sweden. The study was performed according to the Declaration of Helsinki.

### fMRI acquisition

MR imaging was performed using a 3T whole body scanner (Achieva 3T X Philips scanner, Philips Medical Systems, Best, The Netherlands) equipped with an 8-channel head coil. An anatomical T_1_-weighted reference data set to a voxel size of 0.8 × 1.0 × 2.0 mm^3^ and 60 slices was acquired at the beginning of each scanning session. During stimulus presentation, blood oxygen level-dependent (BOLD) imaging was performed using a single-shot echo planar imaging sequence with echo time/repetition time = 35/3000 ms, flip angle = 90°, acquisition matrix = 76 × 77, acquired voxel size = 3.0 × 3.0 × 3.0 mm^3^ and 30 slices.

The participants were lying on their back in the scanner with their head lightly fixated by Velcro strips. During scanning, visual stimuli were presented through goggles mounted on the head coil (Visual System, NordicNeuroLab, Bergen, Norway). The stimulus paradigm was implemented using the commercial software package E-prime (Psychology Software Tools, Sharpsburg, PA, USA). To synchronize the paradigm and the MR-scanner, trigger pulses from the scanner were fed to the paradigm-controlling PC through SyncBox (NordicNeuroLab, Bergen, Norway).

To induce emotional anticipation, we employed a paradigm that has previously been shown to discriminate anticipatory brain reactivity across the hormonal fluctuations of the menstrual cycle^13^. In this paradigm, cued images of positive or negative valence were presented on a screen. Each image was preceded by a color cue indicating the valence of the subsequent image. The color cues were red slides for negatively valenced images and green slides for images of positive valence. The red or green slide was presented for 5 s, immediately followed by a black screen for a duration of 2.5–3.5 s, after which the picture was presented for 2 s. After each color cue-image combination, a black screen was displayed for 9–11 s before the next color cue-image pair appeared. The presentation order sequence was pseudo-randomized. As emotional stimuli, 15 negative and 15 positive images were selected from the International Affective Pictures System (IAPS)^29^. The images were matched for valence and arousal according to the normative ratings in the IAPS material. Arousal ratings was similar for positive and negative images, and the mean valence ratings of positive and negative pictures were clearly separated^13^.

### Preprocessing of fMRI

Data were analyzed in MatLab (MathWorks, Natick, Massachusetts, United States of America) using SPM 5, available at http://www.fil.ion.ucl.ac.uk/spm/software/spm5. BOLD images were realigned to a mean image of each session, slice timed to the middle slice of each whole-brain volume and normalized into MNI-space using normalization parameters obtained from a segmentation of the individual anatomic scan. Smoothing was performed using an 8-mm Gaussian kernel (full-width half-maximum). For each participant, the BOLD signal was convolved with the canonical hemodynamic response function provided by SPM, regressed on the stimulus function (boxcar, onsets and durations of color slides and images (each valence processed separately)), the 2.5–3 s of black screens between each stimulus and six movement parameters obtained from the realignment step. For each individual, contrast maps of the reactivity for anticipation of negative emotional stimuli (operationalized as red color screens contrasted against green color screens) and negative emotion processing (operationalized as negative stimuli contrasted against positive stimuli) were obtained. The contrast maps were then used for second level, random effect group comparisons and correlations.

### Hormonal analyses

Serum progesterone and estradiol levels were analyzed by competitive immunometry electrochemistry luminescence detection at the Department of Clinical Chemistry, Uppsala University hospital. The samples were run on a Roche Cobas e601 with Cobas Elecsys estradiol and progesterone reagent kits, respectively (Roche Diagnostics, Bromma, Sweden). For progesterone, the measurement interval was 0.1–191 nmol/l and 18.4–15781 pmol/l for estradiol. The progesterone intra-assay coefficient of variation was 2.2% at 2.4 nmol/l and 2.8% at 31.6 nmol/l, while the estradiol intra-assay coefficient of variation was 6.8% at 85.5 pmol/l and 2.8% at 1640 pmol/l.

### Statistics

Differences between the early and late postpartum test sessions were analyzed in SPM using paired *T*-tests. A component ROI was created using the AAL-definitions (amygdala, insula and ACC) and TD-labels (Brodmann Areas 6, 8, 9, 10, 44, 45 and 46) from the WFU PickAtlas^30–32^. Seed-based connectivity analyses to the amygdala and insula were also performed at both sessions based on the clusters where significant differences had been found between the early and late postpartum assessment. The statistical threshold for comparisons between the two time-points and for connectivity analyses was set to *p *< 0.01, corrected for volume of ROI, and with an extent threshold of 10 contiguous voxels. Spatial localizations are reported in Talairach coordinates.

Within-subject comparisons of hormonal levels were made by Wilcoxon signed-ranks tests. Correlations between extracted beta-values from the SPM analyses and hormonal levels were performed using Spearman rank correlations. All analyses outside of SPM were performed using SPSS, version 20.0 (SPSS Inc., Chicago, Illinois, USA), with a *p*-value < 0.05 considered significant. All values in the text are displayed as the mean ± standard deviation, unless otherwise stated.
